# Supplementary material for: Unveiling the Neuroprotective Potential of Date Palm (Phoenix dactylifera): A Systematic Review
Source: Pharmaceuticals (Basel). 2024 Sep 17;17(9):1221. doi: 10.3390/ph17091221 (PMC11434792; doi:10.3390/ph17091221)
Supplement: Supplementary file 1 [file pharmaceuticals-17-01221-s001.zip › pharmaceuticals-3167543-supplementary.pdf]

Table S1: Electronic search thread used for *Phoenix dactylifera*.

Literature Search Engine	*Number*	*URL (Uniform Resource Locator/Web Address)*
PubMed	1.	https://pubmed.ncbi.nlm.nih.gov/38687201/
	2.	https://pubmed.ncbi.nlm.nih.gov/?term=phoenix+dactylifera
	3.	https://pubmed.ncbi.nlm.nih.gov/29557751/
	4.	https://pubmed.ncbi.nlm.nih.gov/34929829/
	5.	https://pubmed.ncbi.nlm.nih.gov/36358976/
	6.	https://pubmed.ncbi.nlm.nih.gov/32588917/
	7.	https://pubmed.ncbi.nlm.nih.gov/30054703/
	8.	https://pubmed.ncbi.nlm.nih.gov/35894352/
	9.	https://pubmed.ncbi.nlm.nih.gov/37170935/
	10.	https://pubmed.ncbi.nlm.nih.gov/30906133/
	11.	https://pubmed.ncbi.nlm.nih.gov/35234023/
	12.	https://pubmed.ncbi.nlm.nih.gov/33327616/
	13.	https://pubmed.ncbi.nlm.nih.gov/36014407/
	14.	https://pubmed.ncbi.nlm.nih.gov/35233390/
	15.	https://pubmed.ncbi.nlm.nih.gov/37892156/
	16.	https://pubmed.ncbi.nlm.nih.gov/36100779/

Literature Search Engine	*Number*	*URL (Uniform Resource Locator/Web Address)*
	**17.**	https://pubmed.ncbi.nlm.nih.gov/34834913/
	**18.**	https://pubmed.ncbi.nlm.nih.gov/35161362/
	**19.**	https://pubmed.ncbi.nlm.nih.gov/32798862/
	**20.**	https://pubmed.ncbi.nlm.nih.gov/31368543/
	**21.**	https://pubmed.ncbi.nlm.nih.gov/37841666/
	**22.**	https://pubmed.ncbi.nlm.nih.gov/35597379/
	**23.**	https://pubmed.ncbi.nlm.nih.gov/32737827/
	**24.**	https://pubmed.ncbi.nlm.nih.gov/31343012/
	**25.**	https://pubmed.ncbi.nlm.nih.gov/30175115/
	**26.**	https://pubmed.ncbi.nlm.nih.gov/33915923/
	**27.**	https://pubmed.ncbi.nlm.nih.gov/33854917/
	**28.**	https://pubmed.ncbi.nlm.nih.gov/32474075/
	**29.**	https://pubmed.ncbi.nlm.nih.gov/28702461/
	**30.**	https://pubmed.ncbi.nlm.nih.gov/28588600/
	**31.**	https://pubmed.ncbi.nlm.nih.gov/28127417/
	**32.**	https://pubmed.ncbi.nlm.nih.gov/34837910/
	**33.**	https://pubmed.ncbi.nlm.nih.gov/36834805/
	**34.**	https://pubmed.ncbi.nlm.nih.gov/31456052/

Literature Search Engine	*Number*	*URL (Uniform Resource Locator/Web Address)*
	**35.**	https://pubmed.ncbi.nlm.nih.gov/33921030/
	**36.**	https://pubmed.ncbi.nlm.nih.gov/34885942/
	**37.**	https://pubmed.ncbi.nlm.nih.gov/29377104/
	**38.**	https://pubmed.ncbi.nlm.nih.gov/32677543/
	**39.**	https://pubmed.ncbi.nlm.nih.gov/37333476/
	**40.**	https://pubmed.ncbi.nlm.nih.gov/38201154/
	**41.**	https://pubmed.ncbi.nlm.nih.gov/23587027/
	**42.**	https://pubmed.ncbi.nlm.nih.gov/24279318/
	**43.**	https://pubmed.ncbi.nlm.nih.gov/31115749/
	**44.**	https://pubmed.ncbi.nlm.nih.gov/23948523/
**SCOPUS, Web of Science, and BIOSIS**	**45.**	https://www.researchgate.net/publication/317389921_Date_Palm_Tree_Phoenix_dactylifera_L_Natural_Products_and_Therapeutic_Options
	**46.**	https://www.researchgate.net/publication/379348673_An_Overview_of_Date_Phoenix_dactylifera_Fruits_as_an_Important_Global_Food_Resource
	**47.**	https://www.academia.edu/323840/Biochemical_and_Nutritional_Characterizations_of_Date_Palm_Fruits_Phoenix_dactylifera_L_
	**48.**	https://core.ac.uk/download/pdf/234689529.pdf
	**49.**	https://www.scielo.br/j/cta/a/qQkjczhzr4DTL3wvjCfVqcR/?format=pdf&lang=en

Literature Search Engine	*Number*	*URL (Uniform Resource Locator/Web Address)*
	**50.**	https://www.semanticscholar.org/paper/Phytochemical-characteristics-of-Date-Palm-(Phoenix-Anjum-Bukhat/624f75d95d32d87810f4a02eef6c9206fd6b357f
	**51.**	https://www.ijpsonline.com/articles/phytopharmacological-review-on-date-palm-emphoenix-dactyliferaem.pdf
	**52.**	https://www.academia.edu/56116898/Date_Palm_Phoenix_dactylifera_Novel_Findings_and_Future_Directions_for_Food_and_Drug_Discovery
	**53.**	https://faculty.ksu.edu.sa/sites/default/files/32-ema_2014.pdf
	**54.**	https://www.tandfonline.com/doi/pdf/10.1080/10408398.2010.499824
	**55.**	https://www.semanticscholar.org/paper/Date-(Phoenix-dactylifera-L.)-by-Products%3A-Chemical-Attia-Reda/6eb2c66fda51316666935905e7a4ab0c2ea04345
	**56.**	https://www.phytojournal.com/archives/2022/vol11issue3/PartB/11-3-2-759.pdf
	**57.**	https://www.interesjournals.org/articles/growth-and-yield-responses-of-a-dry-date-palm-phoenix-dactylifera-l-cultivar-to-soil-and-foliar-fertilizers.pdf
	**58.**	https://jppres.com/jppres/pdf/vol9/jppres21.1108_9.6.921.pdf
	**59.**	https://www.pjoes.com/Effects-of-Date-Palm-Phoenix-dactylifera-Seed-Extract-on-Heavy-Metals-Concentrations-in-Carp-Cyprinus-carpio-,61853,0,2.html

**Literature Search Engine**	***Number***	***URL (Uniform Resource Locator/Web Address)***
	**60.**	https://digital.csic.es/bitstream/10261/147647/1/Postprint_2016_ScientHortic_V211_P352.pdf
	**61.**	https://research.amanote.com/publication/MqFH4nMBKQvf0BhiGP84/date-palm--phoenix-dactylifera-l-biotechnology-a-mini-review
	**62.**	https://www.internationalscholarsjournals.com/articles/mineral-study-of-phoenix-dactylifera-l-leaves-by-inductively-fixed-plasma-optical-discharge-spectroscopy.pdf
	**63.**	https://nnpub.org/index.php/FAES/article/view/1811
	**64.**	https://www.ejmanager.com/mnstemps/53/53-1488226594.pdf?t=1517898558
	**65.**	https://www.diva-portal.org/smash/get/diva2:1568272/FULLTEXT01.pdf
	**66.**	https://theijmed.com/index.php/theijmed/article/view/611/pdf
	**67.**	https://e-century.us/files/ijcem/7/3/ijcem1401053.pdf
	**68.**	https://iopscience.iop.org/article/10.1088/1757-899X/368/1/012009/pdf
